# Elongation of spinal cord reactive astrocytes via LRP1/TRK signaling

**DOI:** 10.1038/s41392-026-02660-1

**Published:** 2026-06-01

**Authors:** Francisco Javier Rodriguez-Jimenez, Francisca Selles, Pavla Jendelova, Slaven Erceg

**Affiliations:** 1 Stem Cell Therapies in Neurodegenerative Diseases Lab, Research Center “Principe Felipe, Valencia, Spain; 2 Department of Neuroregeneration, Institute of Experimental Medicine, Czech Academy of Sciences, Prague, Czechia

**Keywords:** Neurological disorders, Regeneration and repair in the nervous system

**Dear Editor,**

Effective regeneration following spinal cord injury (SCI) or the onset of neurodegenerative disease entails the coordinated migration of neurons and the regrowth of long-distance axons. Astrocytes, through their reactivity and structural plasticity, play an opposing role: forming glial scars that may inhibit axonal regeneration yet simultaneously guide regrowth through oriented processes. The elongation and polarization of reactive astrocytes represent indispensable morphological adaptations after SCI^1^, yet the upstream molecular machinery that regulates such changes remains poorly understood. Here, we define a novel LRP1/TrkA/β-Catenin signaling cascade activated by the small molecule FM19G11^2^ as responsible for inducing astrocyte elongation in vivo and in vitro and propose a possible therapeutic target for injured spinal cord repair.

Our previous study described the regenerative effects of FM19G11 treatment following SCI^3^. To more deeply analyze the mechanisms and signaling pathways involved in these regenerative effects, we explored the impact of FM19G11 (a HIF1α pathway modulator with novel functions in regeneration) on astrocytes. FM19G11 administration induced dramatic stretching of GFAP-reactive astrocytes in the regions near the lesion center as early as 2 days post-injury (dpi), with sustained activity until day 7 (Fig. 1a). Morphometric analysis revealed a longer length of protrusions and a significantly larger orientation angle along the lesion axis, indicating greater polarization of astrocytic processes (Fig. 1a). We confirmed these findings by confocal imaging and quantified them in dorsal-to-ventral regions of the injured spinal cord (Fig. 1a).

**Fig. 1:**
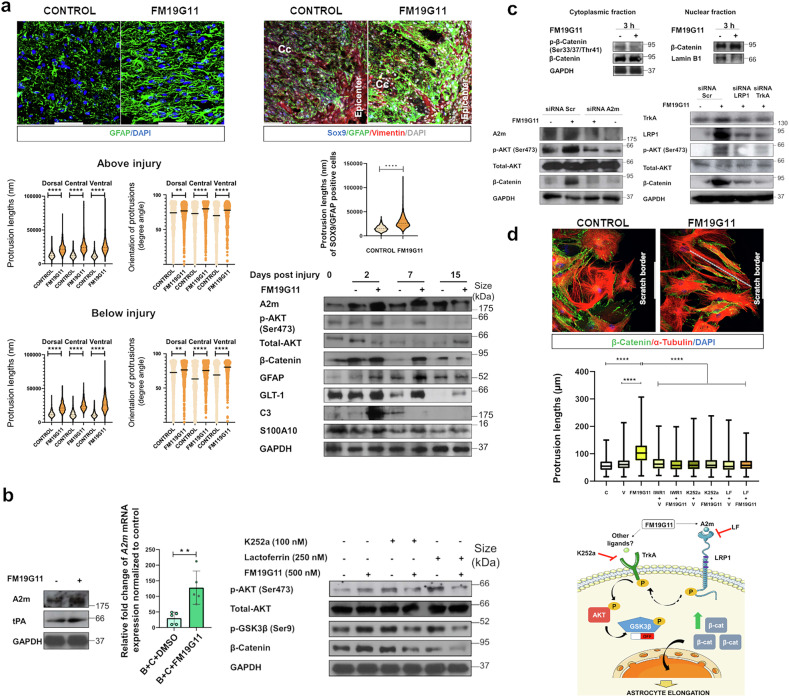
FM19G11 modulates astrocyte morphology and signaling pathways in spinal cord injury and in vitro models. **a** In vivo effects of FM19G11 on injured spinal cord tissue. (Top left) Representative images of GFAP-labeled astrocyte projections above the epicenter at 7 days post-injury (dpi) in control and FM19G11-treated animals. (Scale bars = 50 μm) (Middle and bottom left) Quantification of astrocyte protrusion length and orientation (angle in degrees) above and below the epicenter after 7 dpi demonstrates significant differences between FM19G11-treated and control animals. (*n* = 6, significant differences: Lengths (Above and below injury: ****p < 0.0001); Angles (Above injury): **p = 0,0014, ****p < 0.0001; Angles (Below injury): **p = 0,0025, ****p < 0.0001). (Top right) Central spinal cord sections stained for SOX9 (blue), Vimentin (red), and GFAP (green) near the central canal (CC), with nuclei counterstained with DAPI (white). (Scale bars = 50 µm). (Middle right) Quantification of protrusion length in SOX9⁺/GFAP⁺ cells above the epicenter reveals significant increases in FM19G11-treated animals compared to control animals (*n* = 6, significant differences: ****p < 0.0001). (Bottom right) Western blot analysis of spinal cord tissue at 2, 7, and 15 dpi revealed a significant increase in the LRP1 ligand A2m and downstream effectors: phosphorylated AKT (p-AKT), total-AKT and β-Catenin, as well as in reactive astrocyte markers GFAP, GLT-1, C3, and S100A10 in vivo in FM19G11-treated animals compared to vehicle treated control animals. **b** Analysis of the LRP1/TRK pathway targeted by FM19G11 in vitro in ependymal cells. (Left) Western blot of A2m and tPA in undifferentiated ependymal cells treated with FM19G11 for 90 min revealed an increase in LRP1 ligand A2m, but not tPA. (Middle) This increase also occurred during the induced astrocytic differentiation of ependymal cells with BMP4 (B) and CNTF (C), as shown by RT‒PCR of *A2m* mRNA expression. Expression normalized to *GAPDH* and day 0 (*n* = 5, significant differences: **p = 0,0079). (Right) Western blot of p-AKT, total-AKT, p-GSK3β (Ser9), and β-Catenin in ependymal cells treated with vehicle, FM19G11, or FM19G11 in combination with LRP1 (lactoferrin; LF) or Trk (K252a) inhibitors. **c** Western blot analysis of cytoplasmic and nuclear β-Catenin expression and analysis of the LRP1/TRK pathway after *A2m, LRP1* and *TrkA* siRNA knockdown in ependymal cells targeted by FM19G11. (Top left) Western blot analysis of cytoplasmic β-Catenin stabilization after 3 h of FM19G11 treatment. (Top right) Analysis of nuclear β-Catenin by Western blot, with Lamin B used as the nuclear loading control. (Bottom left) *A2m* knockdown in ependymal cells alters p-AKT, total-AKT, and β-Catenin levels following FM19G11 or vehicle treatment. (Bottom right) *LRP1* and *TrkA* siRNA knockdown blocks FM19G11-induced activation of the LRP1/TrkA signaling pathway in ependymal cells. Western blot analysis showing decreased levels of p-AKT, total-AKT and β-Catenin after siRNA-mediated knockdown. Scrambled siRNA was used as a control. GAPDH was used as loading control. **d** Scratch assay and effect of FM19G11 on mouse astrocytes and schematic model of FM19G11-induced astrocyte elongation via LRP1/TrkA-mediated β-Catenin stabilization. (Top) Representative images of neonatal astrocytes in the scratch assay, showing protrusions (dashed lines) directed toward the wound in vehicle- or FM19G11-treated cells. α-Tubulin (red), β-Catenin (green), and DAPI (nuclei) (Scale bars = 100 µm). (Middle) Quantification of protrusion length (nucleus to tip) in neonatal astrocytes treated with vehicle or FM19G11 ± inhibitors (IWR-1, LF, K252a). (Significant differences: ****p < 0.0001). (Bottom) FM19G11 inhibits GSK3β, promoting β-Catenin accumulation, nuclear translocation, and transcriptional activation through LRP1/TrkA pathway. Pathway-specific inhibitors block these effects. Illustrations were adapted from Servier Medical Art (https://smart.servier.com)

Ependymal cells represent latent neural stem cells in the adult spinal cord that contribute to the generation of astrocytes following injury. To identify the astrocyte subsets that react with FM19G11 in vivo, we performed coimmunostaining for SOX9 and Vimentin (well-known markers for spinal cord ependymal cells) and the astrocytic marker GFAP. As expected, while the percentage of SOX9/GFAP/Vimentin triple-positive astrocytes remained low, we observed an evident population of SOX9/GFAP double-positive astrocytes with significantly longer protrusions following FM19G11 treatment (Fig. 1a). These cells most likely represent ependymal-derived astrocytes or border-forming reactive astrocytes with regenerative potential, as already proposed in scar remodeling. The elongation of this specific astrocyte subset suggests that FM19G11 selectively enhances the morphodynamic features of reactive astrocytes. At the molecular level, we investigated protein expression in vivo in the lesion site and discovered that FM19G11 treatment caused a transient and rapid upregulation of alpha-2-macroglobulin (A2m), a well-known LRP1 ligand^4^, on day 2 (Fig. 1a). This effect correlated with increased AKT phosphorylation and total β-Catenin levels. Concurrently, we observed upregulation of the reactive astrocyte markers GFAP, GLT-1, C3, and S100A10 (Fig. 1a). These findings indicate that FM19G11 induces morphological elongation and elicits a molecular profile characteristic of reactive astrocytes; however, our data do not establish whether this induced reactive state plays a functionally protective or detrimental role. To explore the signaling mechanisms involved, we employed in vitro cultures of mouse spinal cord-derived ependymal cells. FM19G11 treatment caused a transient and rapid upregulation of A2m but not other LRP1 ligands, such as tPA (Fig. 1b). Notably, we observed sustained elevated *A2m* expression throughout the process of ependymal cell differentiation into astrocytes (Fig. 1b). In vitro FM19G11 treatment confirmed LRP1 pathway activation. In parallel with the increase in A2m, FM19G11 markedly activated AKT phosphorylation, induced GSK3β inactivation, and promoted β-Catenin stabilization in ependymal cells (Fig. 1b). Notably, inhibition of LRP1 or the TrkA receptor prior to FM19G11 treatment abolished these actions, indicating that FM19G11 initiates an LRP1–TrkA–AKT–GSK3β–β-Catenin cascade (Fig. 1b). Nuclear/cytoplasmic fractionation confirmed the nuclear translocation of β-Catenin in FM19G11-treated cells, providing direct proof of pathway activation (Fig. 1c). We performed small interfering (si)RNA-mediated knockdown of *A2m*, gene that expresses A2m as LRP1 ligand, in ependymal cells to confirm the requirement of A2m for this signaling pathway, which inhibited AKT phosphorylation and β-Catenin accumulation following treatment with FM19G11 (Fig. 1c), positioning A2m as a necessary upstream effector and LRP1 ligand. Additionally, the siRNA knockdown of LRP1/TrkA confirmed the involvement of this pathway in FM19G11’s mechanism of action (Fig. 1c). These findings demonstrate that FM19G11 initiates intracellular signaling through an A2m-dependent LRP1/TrkA pathway to stabilize and induce the translocation of β-Catenin, a transcriptional coactivator previously reported to influence astrocyte morphology and function. These genetic ablation experiments remain specific to ependymal cells, with their relevance to astrocyte populations remains currently undetermined. We then asked whether functional cytoskeletal responses mirror these molecular changes. In a scratch-wound assay of primary neonatal spinal cord astrocytes, FM19G11 significantly increased the length of astrocytic protrusions toward the scratch border (Fig. 1d). We discovered that LRP1 (lactoferrin; LF), TrkA (K252a), or β-Catenin (IWR-1) inhibitors blocked this effect (Fig. 1d), confirming that FM19G11-induced morphological changes involve the coordinated activation of the LRP1/TrkA/β-Catenin axis.

Combined, these in vivo and in vitro findings validate a mechanistic model in which FM19G11 triggers enhanced A2m expression, which leads to the transactivation of Trk receptors through LRP1 activation. The subsequent receptor interaction leads to AKT phosphorylation, GSK3β inhibition, and β-Catenin stabilization, with subsequent translocation into the nucleus, inducing morphological elongation of reactive astrocytes (Fig. 1d). This cascade supports structural remodeling of astrocytes and is associated with a swift upregulation of reactive astrocyte protein markers (GFAP, GLT-1, C3 and S100A10).

The novelty of our findings lies in the determination of LRP1/TrkA cosignaling as a principal modulator of astrocyte stretching and that FM19G11 selectively targets and stimulates this pathway. To the best of our knowledge, this study represents the first description to link FM19G11 with β-Catenin-dependent cytoskeleton remodeling in astrocytes. The multifaceted ability of FM19G11 to control metabolic and regenerative pathways may render this HIF1α pathway modulator a multitasking tool for central nervous system repair strategies.

Notably, the early occurrence of astrocyte elongation and β-Catenin activation indicates that a therapeutic window could exist within which the injury niche can undergo remodeling to enhance regeneration^5^. Astrocyte process extension toward the lesion may facilitate axonal guidance; such properties may be especially advantageous when combined with cell transplantation therapy or engineered scaffolds that require permissive tissue structure.

In conclusion, we identified a previously unknown mechanism of astrocyte stretching mediated by FM19G11-activated A2m–LRP1/TrkA/β-Catenin signaling. Our data provide mechanistic and therapeutic insights into the regulation of astrocyte morphology and phenotype in SCI, leaving for future research the question of whether pathway modulation may enhance axon regeneration and functional recovery in combination with neuroregenerative treatments.

## Supplementary information

Supplementary Methods

## Data Availability

All data are presented in the main text.
