# Supplementary material for: Elongation of spinal cord reactive astrocytes via LRP1/TRK signaling
Source: Signal Transduct Target Ther. 2026 Jun 1;11:206. doi: 10.1038/s41392-026-02660-1 (PMC13226644; doi:10.1038/s41392-026-02660-1)
Supplement: Supplementary file 1 — Supplementary Methods [file 41392_2026_2660_MOESM1_ESM.docx]

Supplementary Materials for

**Elongation of spinal cord reactive astrocytes via LRP1/TRK signaling**

Francisco Javier Rodriguez-Jimenez, Francisca Selles, Pavla Jendelova and Slaven Erceg.

Correspondence to: [serceg@cipf.es](mailto:serceg@cipf.es)

**This PDF file includes:**

Materials and Methods

Materials and Methods

*Isolation, culture, and FM19G11-treatment of mouse ependymal cells*

Ependymal cells were obtained from the spinal cord of healthy C57BL/6 mice using a previously outlined method ^1^. The acquired ependymal cells were then cultured as neurospheres in a complete medium, following established protocols ^2-4^. The isolated neurospheres were subjected to treatment with either vehicle (dimethyl sulfoxide, DMSO) or FM19G11 (in DMSO) at a concentration of 500 nM for *in vitro* studies. For modulation of specific pathways, drug inhibitors were introduced: K252a (100 nM) to inhibit Trk receptors ^5^ and lactoferrin (LF) (250 nM) to inhibit LRP1 signaling ^6^. These inhibitors were added to the cells 1 hour before FM19G11 treatment.

*Astrocyte-directed differentiation of FM19G11-treated ependymal cells*

Neurospheres underwent a two-week culture period as previously described ^7^. The cells then underwent differentiation into astrocytes using 100% GRP medium supplemented with bone morphogenetic protein 4 (BMP4, B) (10 ng/ml) and ciliary neurotrophic factor (CNTF, C) (20 ng/ml). Throughout the differentiation process, cells were treated with either the vehicle or FM19G11 for a duration of up to fifteen days. Subsequently, cells were harvested for further analysis through quantitative real-time polymerase chain reaction (qRT-PCR) using TaqMan probe for *A2m*.

*RNA isolation and quantitative real-time polymerase chain reaction*

Total RNA extraction from ependymal cells was subjected to reverse transcription using TaqMan reverse transcription was done as it was previously described ^8^. The MGB Assay-on-Demand TaqMan probes employed are *A2m* (Mm00558642_m1). The comparative threshold cycle (Ct) method was employed to determine relative expression.

*Isolation of neonatal mouse cortical astrocytes for primary cultures*

Primary astrocytes were derived from the spinal cords of one-day postnatal mice. Meninges were meticulously dissected from the tissues. Mechanical homogenization of the spinal cord tissue was carried out in high-glucose DMEM supplemented with 20% fetal bovine serum (FBS) using a fire-polished glass pipette and then vortexed for 30 s before passing through a 90 µm filter. The medium was changed every two days, and after one week, the astrocytes underwent a medium change using high-glucose DMEM supplemented with 10% FBS and 1% penicillin/streptomycin.

*Western Blotting analysis*

Cells (n = 4) or spinal cord tissue segments (n = 4) measuring 1 cm at the injury epicenter were collected and Western blot was performed as described ^8^.The specific antibodies included: anti-α2-Macroglobulin (A2m) antibody (PA5-86872; Thermo Fisher Scientific), anti-tPA antibody (SC-515562; Santa Cruz Biotechnology), anti-LRP (26387, Cell Signaling), anti-panTrk (92991 (Cell Signaling), anti-Phospho-AKT (Ser473) antibody (9271; Cell Signaling), anti-AKT antibody (9272; Cell Signaling), anti-Phospho-GSK-3β (Ser9) (5B3) antibody (9323; Cell Signaling), anti-phospho-β-Catenin (Ser33/37/Thr41) antibody (9561; Cell Signaling), anti-β-Catenin antibody (610154; BD Transduction Laboratories), anti-Lamin B1 antibody ab16048; Abcam), anti-GFAP antibody (PA1-10004; Thermo Fisher Scientific), anti-GLT-1 antibody (PA5-19706; Thermo Fisher Scientific), anti-C3 antibody (A13283; ABclonal), anti-S100A10 antibody (ab187201; Abcam), and anti-GAPDH antibody (MAB374; Millipore) used as a loading control. Subsequently, membranes were incubated with rabbit anti-mouse or anti-goat horseradish peroxidase-conjugated secondary antibody (1:10000) (Sigma Chemical). Blots were visualized using the ECL detection system (Amersham), and the results were quantified by densitometry using ImageJ Software.

*Immunohistochemical staining*

Cells or tissues were fixed using 4% paraformaldehyde (PFA) at room temperature for 15 minutes. Following the permeabilization of cell membranes with 0.05% Triton X-100, samples were subsequently blocked with PBS containing 10% FBS. Primary antibodies (1:200) were incubated for immunocytochemical/immunohistochemical evaluation overnight at 4 ºC. Immunohistochemical assessment was conducted two, seven-, and fifteen-days post-injury (dpi). Spinal cord section slides were processed for immunohistochemistry using the following primary antibodies: anti-GFAP antibody (PA1-10004, Thermofisher), anti-SOX9 antibody (702016; Thermofisher), anti-vimentin antibody (ab8978; Abcam). Secondary antibodies included Goat anti-Mouse IgG (H+L), Alexa Fluor™ 633 (A-21050; Invitrogen), Goat anti-rabbit Alexa Fluor™ 555 (A-21428; Invitrogen) Goat anti-Mouse IgG (H+L), Alexa Fluor™ 488 (A-11001; Invitrogen), and Goat anti-Chicken IgY (H+L), Alexa Fluor™ 488 (A-11039; Invitrogen). Incubation with secondary antibodies occurred for 1 hour at room temperature. After additional washing, samples were counterstained with 4,6-diamidino-2-phenylindole (DAPI) for nuclear visualization and cover-slipped for microscopic evaluation. Confocal microscopy (Leica TCS-SP2-AOBS, Wetzlar, Germany) was employed to visualize and analyze the signals.

*Nuclear and cytoplasmic protein fractionation*

Ependymal cells, cultivated as neurospheres, underwent a 3h exposure to the vehicle (DMSO) or FM19G11 (500 nM) to investigate any impact on β-Catenin stabilization and nuclear translocation. Nuclear and cytoplasmic protein fractionation was performed with treated samples using a nuclear extraction kit following the manufacturer's instructions (ab113474; Abcam).

*Transfection of small interfering RNA (siRNA) for the ablation of β-Catenin expression in stem cells*

For siRNA experiments, Ependymal cells grown as neurospheres *in vitro* were dissociated using 0.05% trypsin-EDTA and plated at a density of 200,000 cells per ultra-low attachment 24-well and transfected with two siRNAs of A2m (SASI_MM01_00111690 and SASI_MM01_00111691, Sigma Aldrich), LRP1 (SASI_MM01_00199885, SASI_MM02_00314075, SASI_MM01_00199887) and TrkA (SASI_MM02_00288185, SASI_MM02_00288187, SASI_MM02_00288189), or control siRNA (MISSION® siRNA Universal Negative Control #1, SIC001, Sigma Aldrich) (50 pmol) using 2 µl of Lipofectamine™ RNAiMAX Transfection Reagent (Invitrogen). After 48 h, Lipofectamine the cells were treated with DMSO or FM19G11 (500 nM) for 90 min.

*Scratch assay*

Neonatal spinal cord-derived primary astrocytes were seeded and cultured in poly-L-lysine-coated 6-well chambers. Subsequently, the astrocytes underwent overnight serum starvation. On the following day, prior to treatment with either DMSO (vehicle) or FM19G11 (500 nM), the astrocytes were pre-treated for 1 hour with IWR1 (1 µM) to inhibit β-Catenin (2), K252a (100 nM) to inhibit Trk ^5^, and LF (250 nM) to inhibit LRP1 signaling ^6^. After a 24-hour incubation, a confluent neonatal astrocytic monolayer was scratched using a P10 pipette tip, and detached cells and debris were immediately removed through three washes with PBS. The cells were then maintained for 16 hours in a serum-free culture medium before fixation with 4% PFA for 15 minutes. Immunohistochemical evaluation was performed using α-tubulin monoclonal antibody (Sigma, #T9026, 1:200) and β-Catenin antibody (to delineate the cell membrane) (9587, Cell Signaling, 1:200). Images of astrocyte protrusions were captured using a confocal microscope (Leica TCS-SP2-AOBS, Wetzlar, Germany). The protrusion length of astrocytes, defined as the distance from the tip of the longest protrusion to the nucleus, was quantified using ImageJ software. Approximately 600 cells per condition from three independent experiments were measured.

*Surgical Procedures and FM19G11 treatment in vivo*

Surgical procedures were conducted on female adult C57/BL6 mice, with a weight range of 30-40 g, as previously described ^7^. The animals were treated twice daily with either the vehicle (hydroxypropyl methylcellulose; HPMC) or FM19G11 (in HPMC) (2 mg/kg/day) immediately for two, seven, and fifteen days post-injury (dpi).

*Analysis of elongation in vivo*

Mice (n = 6 per condition) underwent transcardial perfusion with a 0.9% saline solution followed by 4% PFA in PBS. Horizontal serial sections of the spinal cord spanning the injury site were cut at a thickness of 10 µm and utilized for immunohistochemistry. Adjacent sections (three per slide) were spaced 50 μm apart on the same slide. For the horizontal plane, nine serial sections per animal spanning 450 μm of the dorsal-ventral axis of the spinal cord gray matter were subjected to immunohistochemistry at two, seven, and fifteen dpi using an anti-GFAP antibody (PA1-10004, Thermofisher). Protrusion lengths were quantified in a blinded manner. Measurements were taken using the nuclei as the starting point and the furthest protrusion tip as the endpoint. The transverse axis of the lesion served as a reference for the orientation of protrusions with regard to the lesion, where a value of 90° indicated an astrocyte oriented perpendicularly (towards) the lesion core. Five images from above and five from below in the region adjacent to the epicenter of the injury (T8–T9 level) were captured for each section using confocal microscopy (Leica TCS-SP2-AOBS, Wetzlar, Germany). A total of 100 protrusion measurements were performed per animal, resulting in 600 measurements per condition.

*Statistical Analysis*

Statistical analyses were conducted using GraphPad Prism 5 software. For statistical analyses, the Mann–Whitney U test was used to assess differences between control and experimental groups, while the Kruskal–Wallis test was employed for multiple group comparisons. Values presented as mean ± SD of at least three independent experiments, and significance levels are denoted as follows: **p < 0.01 and ****p < 0.0001 for statistically significant differences.

**REFERENCES**

1 Rodriguez-Jimenez, F. J. *et al.* Connexin 50 Expression in Ependymal Stem Progenitor Cells after Spinal Cord Injury Activation. *Int J Mol Sci*. **16**, 26608-26618, (2015).

2 Reynolds, B. A. & Rietze, R. L. Neural stem cells and neurospheres--re-evaluating the relationship. *Nat Methods*. **2**, 333-336, (2005).

3 Moreno-Manzano, V. *et al.* Activated spinal cord ependymal stem cells rescue neurological function. *Stem Cells*. **27**, 733-743, (2009).

4 Rodriguez-Jimenez, F. J. *et al.* Activation of Neurogenesis in Multipotent Stem Cells Cultured In Vitro and in the Spinal Cord Tissue After Severe Injury by Inhibition of Glycogen Synthase Kinase-3. *Neurotherapeutics*. **18**, 515-533, (2021).

5 Shi, Y. *et al.* Ligand binding to LRP1 transactivates Trk receptors by a Src family kinase-dependent pathway. *Sci Signal*. **2**, ra18, (2009).

6 Mantuano, E., Lam, M. S. & Gonias, S. L. LRP1 assembles unique co-receptor systems to initiate cell signaling in response to tissue-type plasminogen activator and myelin-associated glycoprotein. *J Biol Chem*. **288**, 34009-34018, (2013).

7 Rodriguez-Jimenez, F. J. *et al.* Activation of Neurogenesis in Multipotent Stem Cells Cultured In Vitro and in the Spinal Cord Tissue After Severe Injury by Inhibition of Glycogen Synthase Kinase-3. *Neurotherapeutics*, (2020).

8 Rodriguez-Jimenez, F. J., Clemente, E., Moreno-Manzano, V. & Erceg, S. Organized Neurogenic-Niche-Like Pinwheel Structures Discovered in Spinal Cord Tissue-Derived Neurospheres. *Front Cell Dev Biol*. **7**, 334, (2019).
